# Analytical-stochastic model of motor difficulty for three-dimensional manipulation tasks

**DOI:** 10.1371/journal.pone.0276308

**Published:** 2022-10-19

**Authors:** Andrea Lucchese, Salvatore Digiesi, Carlotta Mummolo

**Affiliations:** Department of Mechanics, Mathematics and Management, Polytechnic University of Bari, Bari, Italy; Justus Liebig Universitat Giessen, GERMANY

## Abstract

Multiple models exist for the evaluation of human motor performance; some of these rely on the Index of Difficulty (ID), a measure to evaluate the difficulty associated to simple reaching tasks. Despite the numerous applications of the ID in reaching movements, the existing formulations are functions of the geometrical features of the task and do not consider the motor behaviour of subjects performing repetitive movements in interaction with the environment. Variability of movements, length of trajectories, subject-specific strength and skill, and required interaction with the environment are all factors that contribute to the motor difficulty experienced by a moving agent (e.g., human, robot) as it repeatedly interacts with the environment during a given task (e.g., target-reaching movement, locomotion, etc.). A novel concept of motor difficulty experienced by an agent executing repetitive end-effector movements is presented in this study. A stochastic ID formulation is proposed that captures the abovementioned factors and applies to general three-dimensional motor tasks. Natural motor variability, inherent in the proposed model, is representative of the flexibility in motor synergies for a given agent-environment interaction: the smaller the flexibility, the greater the experienced difficulty throughout the movement. The quantification of experienced motor difficulty is demonstrated for the case of young healthy subjects performing three-dimensional arm movements during which different objects are manipulated. Results show that subjects’ experienced motor difficulty is influenced by the type of object. In particular, a difference in motor difficulty is observed when manipulating objects with different grasp types. The proposed model can be employed as a novel tool to evaluate the motor performance of agents involved in repetitive movements, such as in pick and place and manipulation, with application in both industrial and rehabilitation contexts.

## Introduction

The evaluation of human motor performance is a topic of interest in multiple applications, ranging from manual operations in a production line, to human-computer interface, and human biomechanics and rehabilitation. In these fields, movements such as reaching and pick and place are most commonly studied from the perspectives of motor (e.g., learning, planning, coordination, control [1–3]) and cognitive (e.g., mental workload [4]) performances. Within the motor perspective, measures of difficulty of reaching tasks have been established throughout the decades.

### Background on the index of difficulty

First studies on difficulty measures of a motor task refer to the Fitts’ law and to the related Index of Difficulty (*ID*) [5]. The *ID* was derived by analogy with the Shannon theorem n° 17 [6], theorized within the information theory. The *ID* measures (in bit unit) the difficulty associated with a simple reaching task with target of width *W* placed at a distance *D* from the starting point:

$$ID={\text{log}}_{2}\left(\frac{2\cdot D}{W}\right)$$ (1)

MacKenzie proposed a variation from the original *ID* formulation to improve the model fit on Fitts’ empirical data [7]:

$$ID={\text{log}}_{2}\left(\frac{D}{W}+1\right)$$ (2)

Both formulations refer to simple ‘point-to-point’ reaching tasks that do not consider features of the movement trajectory (shape, length). A generalization of the *ID* formulation to a general planar trajectory *t*, is proposed in [8]:

$$D_{t}=\int_{t}\frac{ds}{W_{t}\left(s\right)}$$ (3)

Here, the trajectory is spatially constrained along the path in a two-dimensional space and is considered as an infinite sequence of simple reaching tasks with target width *W*_*t*_(*s*) that varies at the curvilinear coordinate *s*, and is orthogonal to *t* at *s*.

The above-mentioned *ID* formulations have been applied in reaching tasks considering 3D [9–13] or 2D targets [14, 15], and have been mostly employed in human-computer interaction applications involving the use of devices such as mouse, trackball, joystick, haptic devices, 3D glasses (AR/VR) [8, 15–22].

The existing *ID* models are evaluated a priori, based solely on the pre-defined geometrical features of the task. However, these models are mostly useful when employed as tools for the prediction of valuable subject-specific indicators of motor performance, such as the Movement Time (*MT*) [23, 24] and the “Speed-Accuracy” trade-off [25]. For example, the prediction of *MT* as a function of the *ID* has been done a posteriori, based on experimental observations of subjects performing simple reaching tasks in the shortest possible time [5, 7, 8, 24]. Furthermore, the *ID*_*t*_ has been used to improve the prediction of the “Speed-Accuracy” trade-off [25] observed in constrained reaching tasks experiments (e.g., tunnel-traveling task), by considering the influence of speed of execution and task difficulty on the accuracy of movements at the target [26].

### Task difficulty and motor variability

The above geometry-based ID models alone quantify a “geometrical” task difficulty, where tasks with smaller targets and longer paths are relatively more difficult. However, these models do not directly measure a subject-specific motor performance in the execution of a given reaching task. None of the existing *ID* formulations take into account the motor behaviour of a moving agent (e.g., human, robot) and its interaction with the environment. Variability of movements, length of trajectories executed, subject-specific strength and skill, and required interaction with the environment are all factors that contribute to the motor difficulty experienced by a moving agent as it repeatedly interacts with the environment during a given task (e.g., target-reaching movement, locomotion, etc.). Nevertheless, no single performance measure exists that captures the experienced motor difficulty, while taking into account the above-mentioned factors.

In very few studies, the impact of geometry-based task *ID* on the variability of both kinematics and dynamics parameters has been addressed. The relationship between difficulty and motor performance complexity has been investigated through the analysis of the variability of cyclic force trajectories [27], resulting in an inverse relationship between the *ID* and the complexity of motor behaviour in the force domain. In a different study, it has been observed that in tasks with greater *ID* the overall joint configurations variability is reduced, especially the component of variability that does not affect the task performance (i.e., leaves unchanged the goal-oriented performance variables) [28]; the variable patterns in that case do not represent noise, but the use of equivalent motor solutions in reaching a goal. The existence of multiple solutions to produce the same movement is enabled by the abundance of degrees of freedom (e.g., number of muscles and joints) in the redundant human motor system [29].

While the effect of predefined (geometrical) task difficulty on variability has been partly observed [27, 28], it is still not clear what is the role of motor variability in the evaluation of the motor difficulty experienced by a moving agent as it repeatedly interacts with the environment. This is partially due to the fact that variability stems from different sources, depending on the characteristics of the agent and the type of required interaction, and its role should be addressed according to the specific theoretical and experimental context of interest.

Therefore, when seeking a performance measure indicative of the experienced motor difficulty in repetitive movements, it is necessary to (*i*) clearly identify the aspect of motor variability that is relevant to the present quantification of difficulty and (*ii*) define a novel concept of motor difficulty that is no longer a deterministic function of task geometry but represents the stochastic behaviour of a moving agent executing a repetitive motor task while interacting with the environment.

### Multiple aspects of motor variability

The multiple aspects of motor variability have long been studied from various theoretical and experimental perspectives. The uncontrolled manifold theory [30–32] has been used to demonstrate the co-existence of two components of motor variability in the context of motor learning [33]: one component, which arises from redundancy, does not affect the task-relevant dimension, increases exploration, and facilitates the learning; the other component is a task-relevant variability that should be minimized. Similarly, in the context of optimal feedback control theory [34, 35], Todorov et al. observed that optimal performance is achieved by exploiting redundancy, which explains why in a variety of tasks, variability is not minimized, but it is accumulated in task-irrelevant dimensions. In this perspective, similarly to [28], perturbations from the average trajectory should only be minimized if they interfere with task performance.

Therefore, in multiple contexts it can be seen that two components of variability co-exist and that only the component that does not affect the task performance allows the subject to be more flexible. The task-irrelevant variability is enabled by the motor redundancy, whereas the task-relevant variability is attributed to the noise, and therefore should be minimized by the motor control system.

As seen in the literature above, the exploitation of redundancy can be used for improving learning or, more generally, for enhancing flexibility, depending on the type of task. By the definition assumed here, flexibility is the ability to achieve the same task outcome using different movement solutions: an agent with greater flexibility can generate the same task performance with greater variations of the same movement pattern, as compared to an agent with less flexibility [36]. When observed over small time scales (e.g., on a trial-to-trial basis) and small variations of the environmental constraints, movement variability provides a good window into the degree of flexibility of motor synergies (also called “implicit” flexibility) associated with a given task [36].

Although the above interpretation of motor variability as an indication of flexibility is well acknowledged, it cannot be applied to every possible context: it pertains to the case (as that of the present study) of agents performing repetitive motor tasks on a small time scale and under the same movement strategy (i.e., the movement pattern is not “qualitatively” altered, given the minimal environmental changes), during which task-irrelevant variability can be accumulated up to a certain optimal amount [37]. In this context, flexibility has a positive connotation, since it is opposed to the case of overly-rigid systems that are less adaptable to perturbations [37], and can be achieved by channeling the natural movement variability (i.e., variability observed within the same movement, without any externally imposed perturbations) [36]. For instance, it has been shown that while walking on a treadmill, humans exploit redundancy by adjusting stepping movements while maintaining performance: in this perspective the flexibility allows to perform the locomotion task with a level of motor variability that is potentially beneficial [38].

In many other cases, too much or too little variability is both “bad” [37], which suggests a trade-off between flexibility and variability [36]. For instance, movements of unhealthy subjects can sometime show greater variability [39], other times smaller variability [40], as compared to healthy ones, depending on the pathology and experimental conditions. Moreover, the relationship between variability and flexibility cannot easily be assumed during learning, when the amount and structure of movement variability and task performance all change with practice [36, 41].

The present study does not consider the presence of motor disease nor learning and, similar to [28, 34, 35], focuses on repetitive end-effector movements in which the agent can be more flexible and explore equivalent motor solutions (i.e., variations of the same movement pattern) in phases where accuracy is not required, while succeeding in task goals. In this context, a greater motor variability is associated to a greater flexibility (and potentially greater adaptability to perturbations [37]) of the motor control system to manage the abundance of degrees of freedom available to execute a given task in a specific environment. Within the described context of interest, a novel concept of difficulty in motor tasks is described in the following section.

### Experienced difficulty in motor tasks

The novel concept of motor difficulty experienced by an agent performing repetitive motor tasks represents how much the flexibility of movements is limited by both the agent and the environment features. In general, flexibility is limited by the presence of several constraints that can be relative to the specific agent (internal constraints) or to the task/environment (external constraints) [42]. Both types of constraints affect the movement execution, limiting the exploitation of motor redundancy and the spatial configurations that an agent can achieve. When an agent’s movement is more (or less) limited due to internal/external constraints, the difficulty experienced in the task execution should be higher (or lower).

This concept of motor difficulty can be expressed by the stochastic motor behavior of an agent (e.g., human, robot) that arises during a desired interaction with the environment, quantified by the novel stochastic Index of Difficulty. The stochastic behavior of agents performing any repetitive motor tasks, as enabled by the redundant motor system, implicitly and simultaneously reflects the characteristics of the agent (e.g., motor redundancy, strength, skill) and the type of interaction (e.g., task and environmental features).

A task executed with low motor difficulty represents a type of agent-environment interaction that allows greater flexibility in the choice of equivalent motor solutions; in this case, the stochastic behavior on the end-effector is characterized by greater natural variability, calculated relative to an average trajectory throughout the entire path. Vice versa, a greater motor difficulty is associated to limited flexibility of motor synergies in the task execution, quantified by the small variability of the end-effector trajectories throughout the movement.

Limited flexibility (and great difficulty) could be due to external constraints that restrain end-effector movements, hence requiring great accuracy in the control of its position (analogously to the case of reaching small and distant targets, with great geometrical ID). On the other hand, the source of limited flexibility (and great difficulty) could come from internal constraints that make the agent overly rigid and less adaptable to potential perturbations. Lastly, a reduced flexibility could be the result of a particular agent-environment coupling that exhibits a more rigid (hence, difficult) behaviour as compared to one that is assumed to be optimal with respect to some criterion (e.g., resilience, speed, success rate).

The proposed stochastic model should be applied for the evaluation of experienced difficulty within the same task domain and movement strategy, in order to (a) compare the performance of different agents (or populations) executing the same movement pattern or (b) quantify the effects of small environmental changes on the flexibility (and difficulty) of a certain agent (or population) executing a given task.

An example of the first type of application (a) has been presented in a previous work by the authors [43], in which the locomotion performance of young and elderly human subjects has been compared at different speed levels. A preliminary two-dimensional formulation of the stochastic Index of Difficulty was used to show that, in the sagittal plane, the healthy elderly population exhibits throughout the gait cycle a lower motor variability at the swing foot end-effector, as compared to the control population (i.e., young healthy). The elderly motor flexibility is therefore further limited by age-related internal constraints, resulting in a greater experienced motor difficulty when performing the same walking task, especially at higher speed levels.

The second type of application (b) of the stochastic Index of Difficulty is the object of this study, where the experienced difficulty associated with the three-dimensional repetitive manipulation of different objects is quantified for a given agent (healthy young human subjects). It is hypothesized that the flexibility of motor synergies within the same movement pattern is affected by the small changes in the external constraints represented by the handling of diverse objects.

Lastly, the proposed model can also be applied in the future to a third scenario (c), in which the observed motor difficulty of a reference agent-environment pair (e.g., healthy human subject on a staircase) is used as a benchmark of motor performance representative of the desired degree of flexibility in the specific task (e.g., stair climbing), against which the performance of different agent-environment coupling (e.g., exoskeleton-wearing subject on a steeper staircase) can be evaluated.

## Materials and methods

In this section, the stochastic model of experienced motor difficulty is presented for a general three-dimensional repetitive movement of an agent’s end-effector (hand). In the next section, this model will be applied to a dataset of healthy subjects performing three-dimensional hand movements during which different objects are manipulated.

### Stochastic index of difficulty for a general three-dimensional motor task

To quantify the motor difficulty in a repetitive agent-environment interaction, the proposed stochastic model of the Index of Difficulty *ID*_*obs*_ is based on a formal analogy with *ID*_*t*_ (Eq 3), as follows:

$$ID_{obs}\left(s^{*},\varphi \right)=\int_{0}^{s^{*}}\frac{ds}{W_{obs}\left(s,\varphi \right)}$$ (4)

where *W*_*obs*_(*s*, *φ*) is named the “stochastic width” and stores information about the variability of end-effector position at the curvilinear coordinate *s* observed over *n* repeated trajectories *t*_*k*_, *k* = 1, …, *n*, whose average is $\bar{t}$. Assuming a probability density function for the stochastic position of the end-effector, evaluated at a probability level *φ*.

The inverse function of *W*_*obs*_(*s*, *φ*) is integrated with respect to the infinitesimal length *ds* of the average trajectory, over a range of the curvilinear coordinate *s* from zero (initial position) to $s^{*}\in \left[0;\bar{t}\right]$. Nevertheless, *ID*_*obs*_ is not simply the inverse of motor variability, but a cumulative measure that takes into account both the variability of the movement pattern throughout the entire motion and the length of the path. For instance, two agents that perform a repetitive motor task with the same overall movement variability, but along different average trajectories, will experience different motor difficulties.

Furthermore, *ID*_*obs*_ captures the overall flexibility of motor synergies (in the context previously explained) through a cumulative measure of variability, which differs from the typical measures of used in reaching task experiments, where a local form of motor variability is considered (i.e. at specific points, such as target) [44–47].

### Model of stochastic width

**Fig 1:**
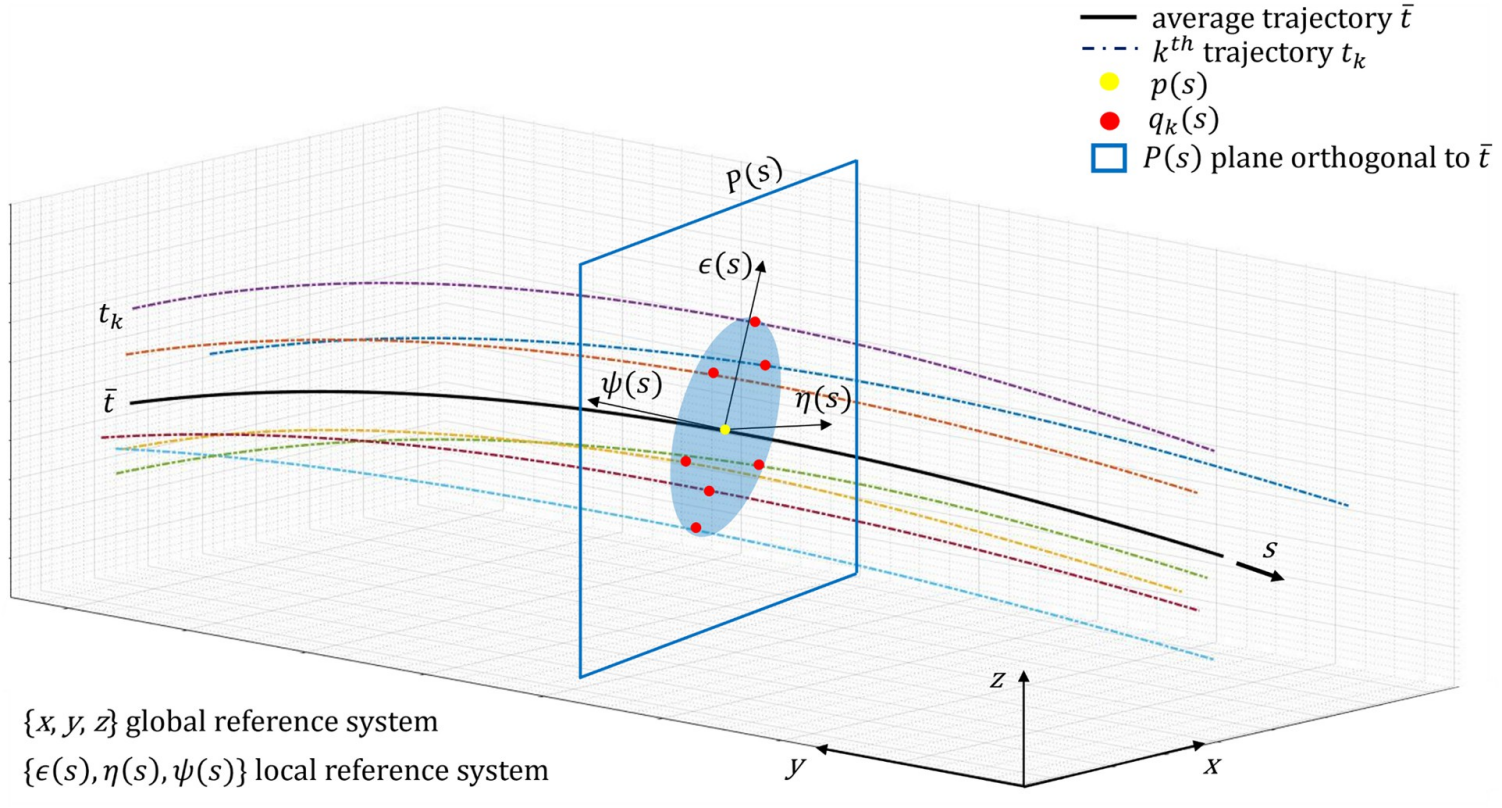
Example of trial trajectories (dash-dotted lines) and their average (thick black line). The light blue ellipse shows the motor variability in the plane P(s) orthogonal to $\bar{t}$ at s.

To account for the motor variability in *ID*_*obs*_(s*, *φ*) throughout the three-dimensional manipulation task, the variability of *n* repeated trajectories is evaluated in the plane orthogonal to the average trajectory $\bar{t}$ at curvilinear coordinate *s* (Fig 1), consistently with the approach of [8, 48].

Each point *q*_*k*_(*s*) (*k* = 1, …, *n*) is the intersection between the trial trajectory *t*_*k*_ and the plane *P*(*s*) orthogonal to the average trajectory at *s*. In the case of bivariate normal distribution of the *n* points *q*_*k*_(*s*), their spatial distribution on *P*(*s*) is expressed by the standard deviation ellipse (light blue area in Fig 1), as observed in field studies [46, 49, 50]. The point *q*_*k*_(*s*), belonging to the average trajectory $\bar{t}$ at curvilinear coordinate *s*, is the centre of the standard deviation ellipse.

The elliptical shape of the spatial distribution of the *n* points *q*_*k*_(*s*) is evaluated by the Principal Component Analysis (PCA) technique [40, 51–53]. The PCA technique is used to identify the principal components (PC) representing the directions of spatial variability of the dataset considered. By applying the PCA at each curvilinear coordinate *s*, a local reference system is identified by the independent variables *ϵ*(*s*), *η*(*s*), *ψ*(*s*) (Fig 1). These variables represent the directions of maximum variance: *σ*_*ϵ*_ and *σ*_*η*_ are the standard deviations of the *n* points *q*_*k*_(*s*) along the first and second PC, *ϵ*(*s*) and *η*(*s*), respectively. Given that all the *n* points *q*_*k*_(*s*) lay in the plane *P*(*s*) orthogonal to $\bar{t}$ at *s*, the standard deviation of the third PC, *ψ*(*s*), is null (*σ*_*ψ*_ = 0) (Fig 1). Under the assumption that at each value of the curvilinear coordinate *s*, the *n* points *q*_*k*_(*s*) are normally distributed along directions defined by *ϵ*(*s*) and *η*(*s*), the sum of squares of the normalized variables ($z_{\epsilon }^{2}\left(s\right)={\left(\frac{\epsilon \left(s\right)}{\sigma_{\epsilon }\left(s\right)}\right)}^{2}$ and $z_{\eta }^{2}\left(s\right)={\left(\frac{\eta \left(s\right)}{\sigma_{\eta }\left(s\right)}\right)}^{2}$), are equal to a constant squared distance *c*^2^:

$$z_{\epsilon }^{2}\left(s\right)+z_{\eta }^{2}\left(s\right)={\left(\frac{\epsilon \left(s\right)}{\sigma_{\epsilon }\left(s\right)}\right)}^{2}+{\left(\frac{\eta \left(s\right)}{\sigma_{\eta }\left(s\right)}\right)}^{2}=c^{2}$$ (5)

which can be rewritten as follows:

$${\left(\frac{\epsilon \left(s\right)}{\sqrt{c^{2}}\cdot \sigma_{\epsilon }\left(s\right)}\right)}^{2}+{\left(\frac{\eta \left(s\right)}{\sqrt{c^{2}}\cdot \sigma_{\eta }\left(s\right)}\right)}^{2}=1$$ (6)

Eq 6 represents an ellipse with semi-axes $\sqrt{c^{2}}\cdot \sigma_{\epsilon }\left(s\right)$ and $\sqrt{c^{2}}\cdot \sigma_{\eta }\left(s\right)$. The area contoured by the ellipse’s equation represents the local variability of the end-effector position; since variability varies based on the confidence region considered (i.e., probability level *φ*), the ellipse must be consequently adjusted. This information in inherent in *c*^2^: by choosing a specific value of *c*^2^ the semi-axes can be properly modified to consider an ellipse that expresses the variability for the chosen probability level. Depending on the number of samples considered to evaluate the motor variability, *c*^2^ is differently calculated [54, 55]. If the entire population is known, or a large sample size is provided, *c*^2^ is expressed by the squared Mahalanobis distance (*MD*^*2*^) and evaluated through the critical values of the Chi-squared distribution with *v*_1_ degrees of freedom ($\chi_{\nu_{1},\varphi }^{2}$). *ν*_1_ is equal to the dimensionality of the motor variability: in the current case of three-dimensional movement, the local variability on *P*(*s*) has dimension *ν*_1_ = 2. By assuming that the entire population is known and choosing a probability level *φ* = 0.95 (95%), the value $\chi_{2,0.95}^{2}=5.992=MD^{2}=c^{2}\left(0.95\right)$ can be obtained from the Chi-squared distribution tables. Nevertheless, for the present dataset, the number of trials executed by each participant in each condition (*n* = 7), are not enough to evaluate the 95% of motor variability by considering *MD*^*2*^. Therefore, when the entire population is not known and a limited number of samples is collected, *c*^2^ is expressed by the Hotelling’s T-squared distance (*T*^2^), and evaluated as the following:

$$T^{2}=\frac{\left(n-1\right)\cdot \nu_{1}}{n\cdot \left(n-\nu_{1}\right)}\cdot F_{\nu_{1},\nu_{2},\varphi }$$ (7)

where $F_{\nu_{1},\nu_{2},\varphi }$ is the critical value of the F-distribution obtained from the F-distribution tables with *ν*_1_ degrees of freedom of the numerator (*ν*_1_ = 2), *ν*_2_ degrees of freedom of the denominator (equal to *n* − *ν*_1_), and probability level *φ*. By considering *φ* = 0.95 (95%), *ν*_1_ = 2, *ν*_2_ = 5 (*n* = 7), from the F-distribution tables *F*_(2,5,0.95)_ = 5.786, and consequently *T*^2^ = 1.984 = *c*^2^(0.95). Therefore, the major semi-axis of the standard deviation ellipse has length $\sqrt{1.984}\cdot \sigma_{\epsilon }\left(s\right)$ and the minor semi-axis has length $\sqrt{1.984}\cdot \sigma_{\eta }\left(s\right)$. By considering the interval θ ∈ [0, 2π], parametrization of Eq 6 leads to:

$$\left\{\begin{array}{c} \epsilon \left(\theta ,s,\varphi \right)=c\left(\varphi \right)\cdot \sigma_{\epsilon }\left(s\right)\cdot \text{cos}\left(\theta \right)\\ \eta \left(\theta ,s,\varphi \right)=c\left(\varphi \right)\cdot \sigma_{\eta }\left(s\right)\cdot \text{sin}\left(\theta \right) \end{array}\right.$$ (8)

The distance between the center of the ellipse, *p*(*s*), and a point on the ellipse can be obtained as:

$$r\left(\theta ,s,\varphi \right)=\sqrt{{\left(c\left(\varphi \right)\cdot \sigma_{\epsilon }\left(s\right)\cdot \text{cos}\left(\theta \right)\right)}^{2}+{\left(c\left(\varphi \right)\cdot \sigma_{\eta }\left(s\right)\cdot \text{sin}\left(\theta \right)\right)}^{2}}$$ (9)

The mean value of the above variable radius of the ellipse, evaluated over the interval θ ∈ [0, 2π] at a probability level *φ*, is:

$$r_{mean}\left(s,\varphi \right)=\frac{1}{2\pi }\int_{0}^{2\pi }r\left(\theta ,s,\varphi \right)\;d\theta$$ (10)

With this integral, the elliptical area that characterizes the spatial distribution at *s* of the *n* points *q*_*k*_(*s*) with probability level *φ* is converted into a circular area of radius *r*_*mean*_(*s*, *φ*). This average radius is used in this study to model the stochastic width associated with the repetitive movement, as follows:

$$W_{obs}\left(s,\varphi \right)=2\cdot r_{mean}\left(s,\varphi \right)$$ (11)

Through this model, the information of the observed end-effector spatial variability on the plane *P*(*s*) can be captured by the single value of the stochastic width *W*_*obs*_(*s*, *φ*). The practical use of the above model of stochastic width *W*_*obs*_(*s*, *φ*) in capturing the motor variability will be shown in the following sections. Nevertheless, other models for stochastic width could be assumed, without losing generality in the proposed analytical-stochastic framework for the evaluation of motor difficulty.

## Results and discussion

The application of the proposed model of difficulty (*ID*_*obs*_) to capture the effects of different external constraints on an agent’s experienced difficulty is demonstrated through the analysis of repetitive movements in the execution of a three-dimensional manipulation task. Results of the statistical analysis on *ID*_*obs*_(s*, *φ*) values are provided and discussed.

### Application of the difficulty model to a manipulation task

The task taken into account consists of reaching, grasping, transporting, and releasing different objects placed at fixed locations on a plane, which represent the external constraints influencing the agent-environment interaction.

An experimental dataset publicly available in [56] is used to test the model. Data analysis is carried out using MATLAB^®^ software (version R2021a). Data refer to 31 healthy individuals; all subjects belong to the same population of young healthy adults and, therefore, no between-subjects differences are assumed. Each individual sits in front of a table to perform the manipulation task with the dominant hand. The initial object position is placed 300 mm from the edge of the table on the same side of the dominant hand; here, the subject grasps an object and moves it to the release position, placed 500 mm symmetrically on the other side of the table, where the object is released and, finally, repositions the hand at the starting point. For the experiment, five different objects have been considered: a tennis ball (105 mm diameter), two one-litre water bottles (80 mm diameter), one full and the other half-full, two ellipsoid shaped balls (62 mm maximum diameter), one soft and the other of stiff plastic. Reflective markers have been placed on the subjects’ end-effector to obtain, through a motion capture system, kinematics parameters of the movement. In the present article, the marker on the hand’s palm is considered for the end-effector movements. Further information on the dataset are in [57].

**Fig 2:**
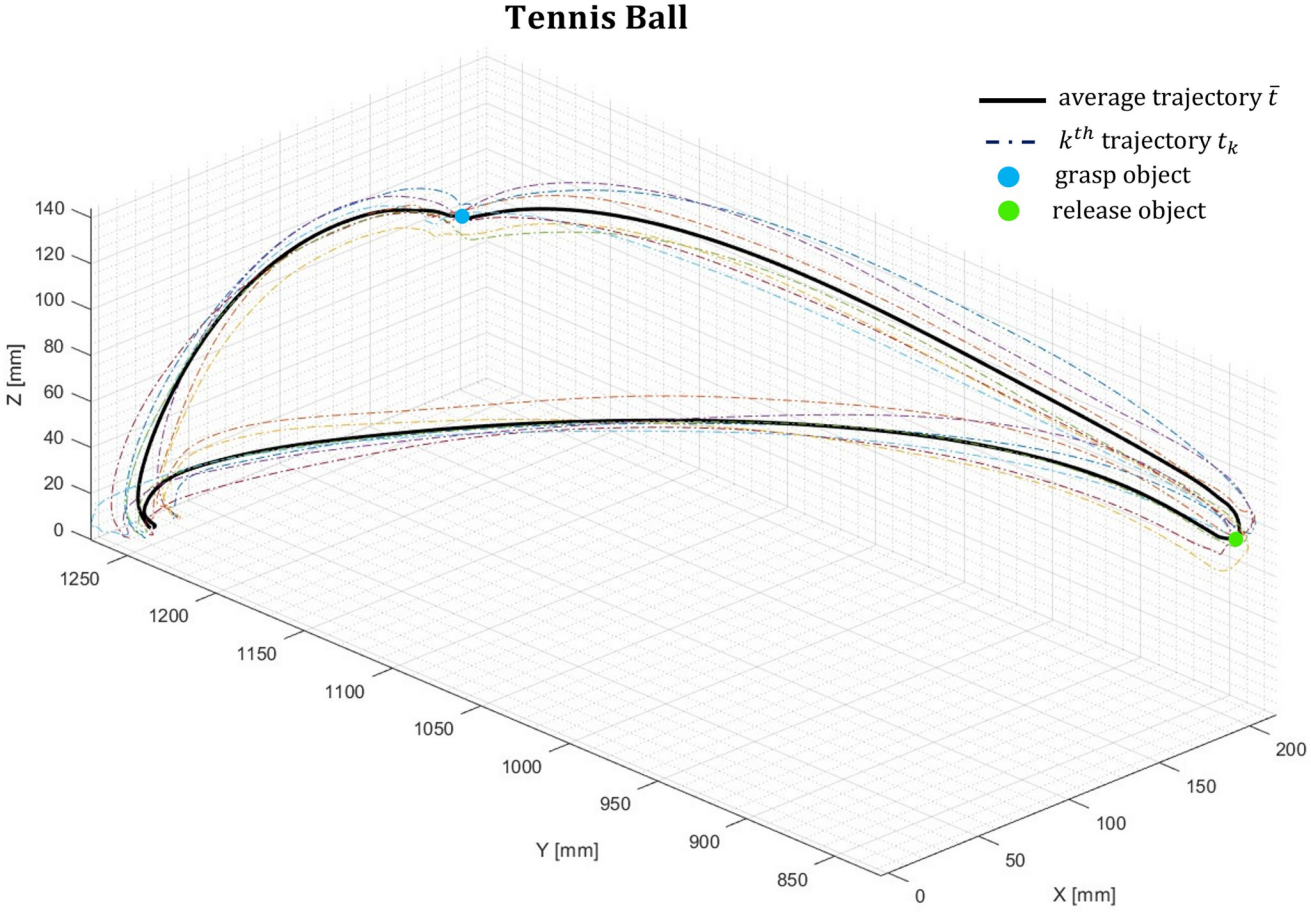
Trial trajectories (dash-dotted lines) and average trajectory (thick black line) for a given subject during the tennis ball manipulation task.

The subjects’ end-effector average trajectory is evaluated on 7 trial trajectories performed during the motor task, for each of the five objects. An example of the trial trajectories of the end-effector (dash-dotted lines) and their average (thick black line) is shown (Fig 2) for a given subject during the manipulation task of the tennis ball.

**Fig 3:**
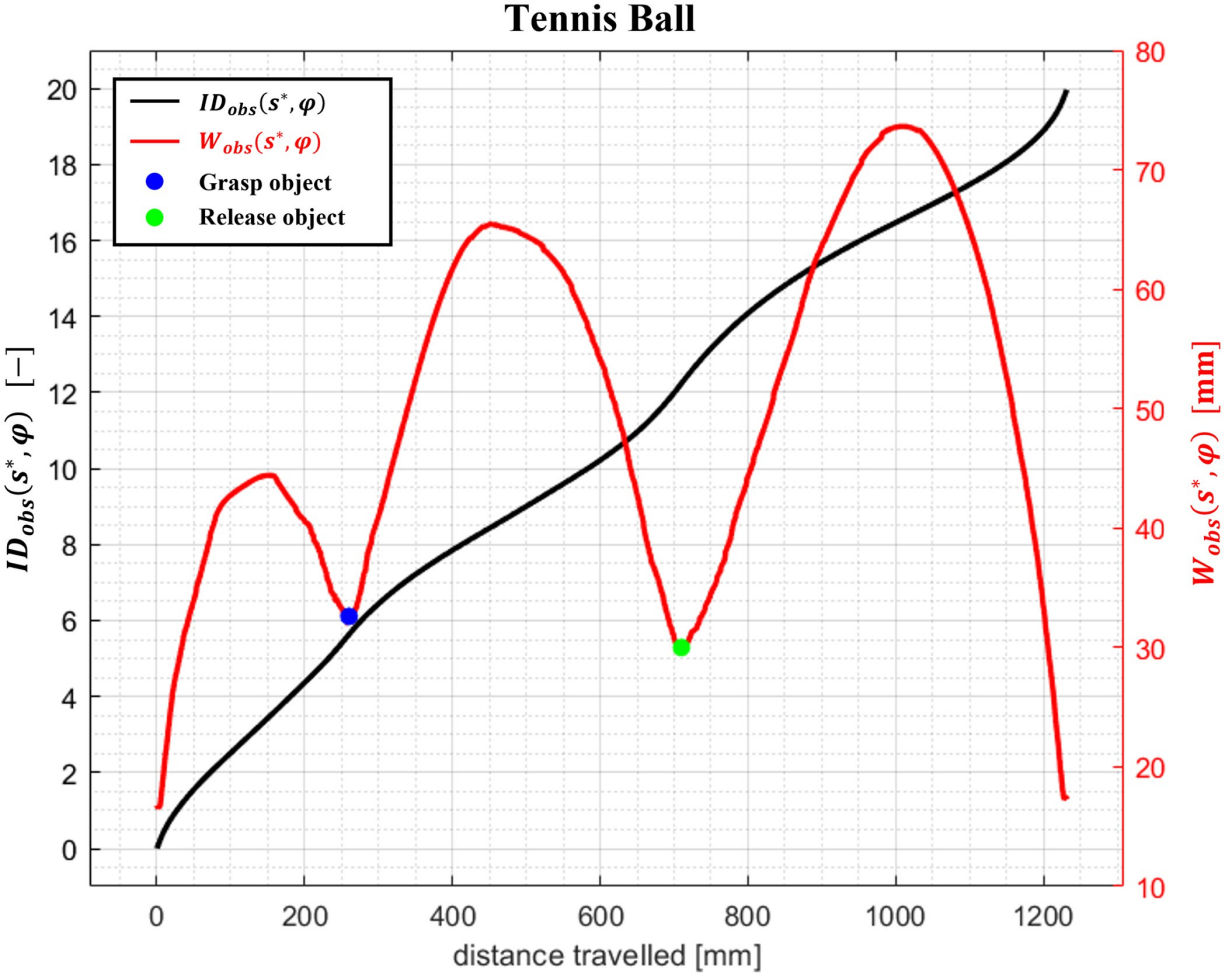
ID_obs_(s*, φ) and W_obs_(s*, φ) values averaged over 31 subjects during the tennis ball manipulation task.

For a given object, Eqs 4 and 11 are applied to the trial trajectories of each subject to determine the values of *W*_*obs*_(s*, *φ*) and *ID*_*obs*_(s*, *φ*), respectively, with *φ* = 0.95 and for s* evaluated from zero (initial position) to the final rest position ($s^{*}=\bar{t}$). As an example, the profiles of the stochastic width and stochastic Index of Difficulty, averaged over the 31 subjects, are shown for the object tennis ball (Fig 3).

The trend of *W*_*obs*_(s*, *φ*) highlights three phases, each of them characterized by a travelling action (gross movement) and a positioning action (fine movement). The first phase (reaching phase) consists of moving the end-effector (hand’s palm) from the starting point to the grasping position; the second phase (transport phase) consists of moving the object from the grasping position and releasing it at the releasing position; finally, the third phase (return phase) consists of moving the empty hand from the releasing point to the starting point where the cycle ends (final placement). For each phase, the trend of *W*_*obs*_(s*, *φ*) is bell-shaped, with smaller values at points where a greater accuracy of the end-effector movements is required (positioning action). These results are in line with [34] since *W*_*obs*_(s*, *φ*) values are higher in the task-irrelevant dimensions (travelling action of each phase), and reach local minima in the task-relevant dimensions (positioning action of each phase) where greater accuracy is required. The exploitation of motor redundancy is greater in the task-irrelevant dimensions since a greater motor variability is observed, witnessed by the large *W*_*obs*_(s*, *φ*) values. At points where accuracy is required, the environment forces the agents to reach a specific target. In short, each phase is characterized by a travelling action where the agent’s end effector is free to explore the space with greater spatial configurations, followed by the positioning action where accuracy is required, resulting in minima values of *W*_*obs*_(s*, *φ*). At points where accuracy is required, the lowest values of *W*_*obs*_(s*, *φ*) cause steeper slopes of the *ID*_*obs*_(s*, *φ*) trend resulting in a greater increase of the motor difficulty.

### Statistical analysis on the stochastic index of difficulty

Different analyses have been performed to test the following two main hypotheses:

1. The manipulation of different objects represents a change in the external constraints, affecting the agent-environment interaction, and influencing the motor difficulty experienced by the subjects during the entire manipulation task. To test the hypothesis, a one-way repeated measures ANOVA on $ID_{obs}\left(s^{*}=\bar{t},\varphi \right)$ values of the 31 subjects has been performed for each condition (i.e., each object). Results show that there is a significative change in the subjects’ motor difficulty when manipulating different objects.
2. In [57] it has been observed that objects with different geometry affect the subjects’ motor behaviour during the object manipulation. To test this hypothesis, a one-way repeated measures ANOVA on $ID_{obs}\left(s^{*}=\bar{t},\varphi \right)$ values of the 31 subjects for the three geometrical different objects (tennis ball, half-full bottle, plastic ellipsoid ball), has been performed. Results show that differences in the motor behaviour, expressed by $ID_{obs}\left(s^{*}=\bar{t},\varphi \right)$, are not associated to the different geometry, but to the different grasp type.

**Fig 4:**
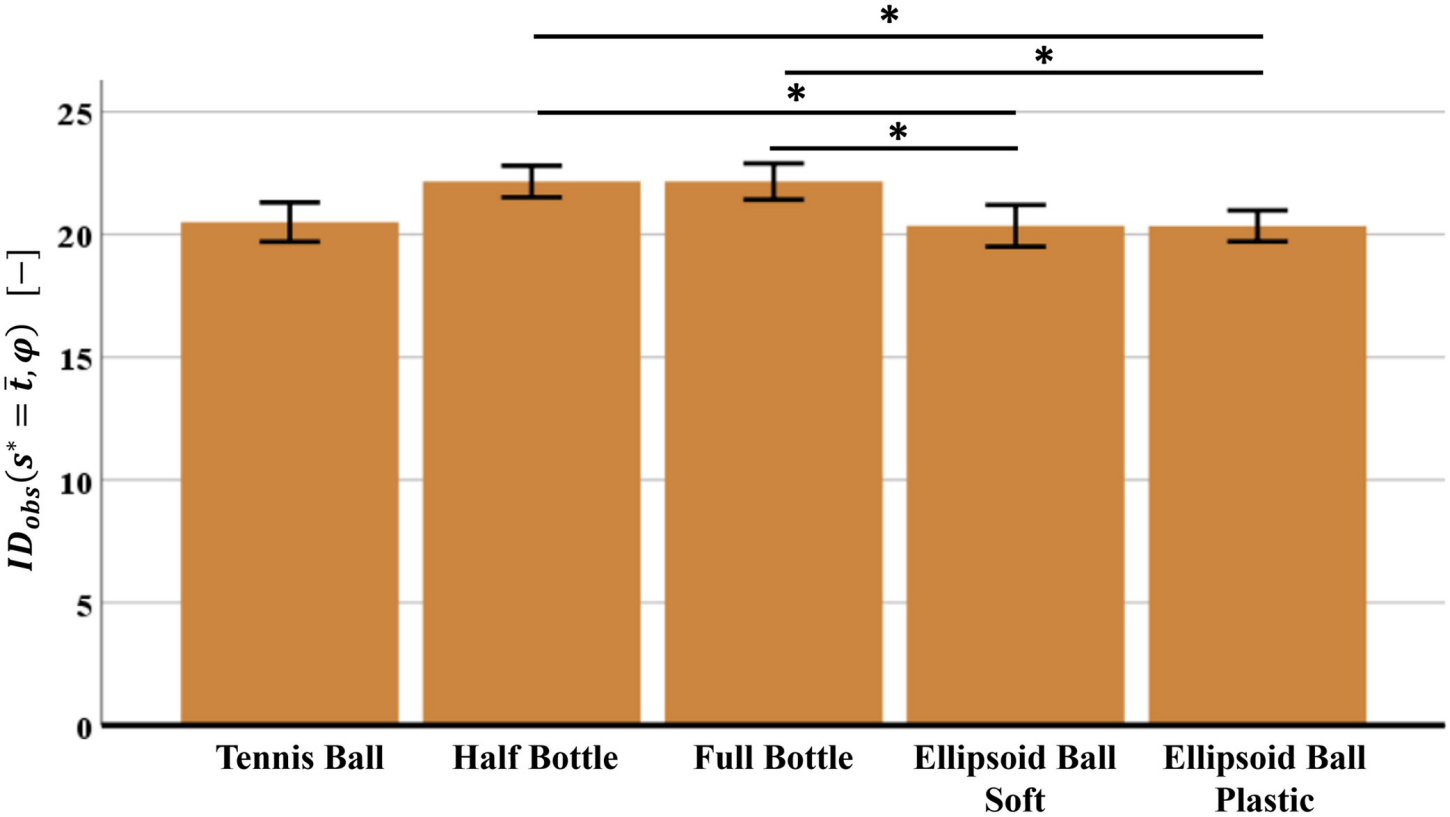
Mean values and SEM (Standard Error of the Mean) of the stochastic Index of Difficulty $ID_{obs}\left(s^{*}=\bar{t},\varphi \right)$, evaluated for the entire manipulation task and for different objects. Asterisks indicate pairwise significant differences (p-value < 0.005); critical p-value = 0.05/10 = 0.005.

To verify whether the five different objects determine a relevant difference on the overall experienced difficulty in the manipulation task (quantified by $ID_{obs}\left(s^{*}=\bar{t},\varphi \right)$), a statistical analysis has been performed using IBM SPSS Statistics^®^ software (version 26). Assumption prior to the one-way repeated measures ANOVA for the $ID_{obs}\left(s^{*}=\bar{t},\varphi \right)$ values of the 31 subjects (normality through Shapiro-Wilk test, sphericity through Mauchly’s test) have been confirmed for all the five objects. ANOVA results show statistically significant differences by comparing the $ID_{obs}\left(s^{*}=\bar{t},\varphi \right)$ values of different objects (*F*(4,120) = 4.361, *p* < 0.003). By applying the post hoc test with Bonferroni correction, the statistically significant difference of $ID_{obs}\left(s^{*}=\bar{t},\varphi \right)$ is evident in case of ellipsoid balls (soft and plastic) vs. bottles (half-full and full) (*p* < 0.005). Results are in Fig 4.

These results confirm the first hypothesis: the proposed stochastic Index of Difficulty is sensitive to capture the different motor difficulty experienced by the population while executing a motor task (object manipulation) with different external conditions (objects). In particular, subjects experience a greater motor difficulty when manipulating the bottles (half-full and full), compared to the ellipsoid balls (soft and plastic). Since results do not provide relevant differences between the two bottles (half-full vs. full), and between the two ellipsoid balls (soft vs. plastic) it can be concluded that the different mass alone as well as, the different stiffness of objets do not influence the population’s motor difficulty; the same conclusion holds in [57] where it has been observed that the alteration of the mechanical properties (weight variations in the bottles, stiffness and friction in the ellipsoid ball), does not affect the motor behaviour. Therefore, the mass end stiffness can be removed from the features that affect the subjects’ motor difficulty.

**Fig 5:**
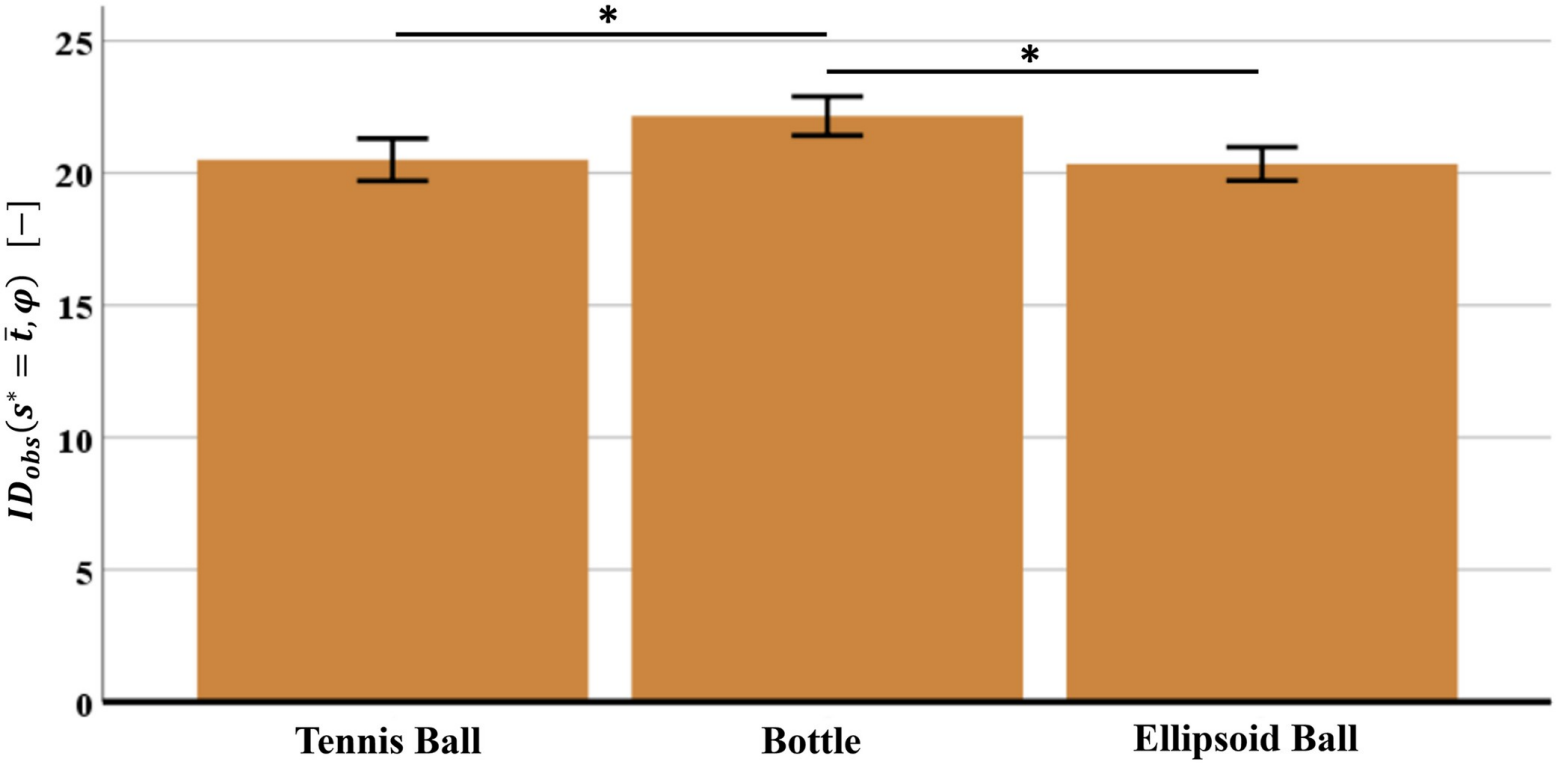
Mean values and SEM (Standard Error of the Mean) of the stochastic Index of Difficulty $ID_{obs}\left(s^{*}=\bar{t},\varphi \right)$ evaluated for the entire manipulation task and for three objects with different geometry. Asterisks indicate pairwise significant differences (p-value < 0.016); critical p-value = 0.05/3 = 0.016.

A further investigation can be carried out focusing only on objects characterized by different geometry (tennis ball, half-full bottle, plastic ellipsoid ball). The following analysis is aimed at verifying if the different geometry is the main object’s feature that affects the subjects’ motor difficulty. Assumption prior to the one-way repeated measures ANOVA for the $ID_{obs}\left(s^{*}=\bar{t},\varphi \right)$ values of the 31 subjects (normality through Shapiro-Wilk test, sphericity through Mauchly’s test) have been confirmed for all the geometrically different objects. ANOVA results show statistically significant differences by comparing the $ID_{obs}\left(s^{*}=\bar{t},\varphi \right)$ values of different objects (*F*(2,60) = 4.010, *p* < 0.02). By applying the post hoc test with Bonferroni correction (*α* = 0.05/3 = 0.016), the statistically significant difference of $ID_{obs}\left(s^{*}=\bar{t},\varphi \right)$ is evident in case of ellipsoid/tennis ball vs. bottle (*p* < 0.001). Results are in Fig 5.

These results do not support the hypothesis that the geometry is the main feature that affects the subjects’ motor difficulty. Nevertheless, statistical differences can be associated with the grasp type of the objects: the tennis ball (spherical) and ellipsoid ball (ellipsoid) are gripped with the same type of grip (’power sphere’ grip), while the bottle (cylinder) is gripped with the ‘large diameter’ grip [58, 59]. No relevant differences are observed by comparing objects grasped with the “power sphere” grip (tennis ball vs. ellipsoid ball); on the contrary, relevant differences are observed when comparing objects with different type of grips, i.e. tennis/ellipsoid ball (“power sphere” grip) vs. bottle (“large diameter” grip) [58, 59]. When differentiating the objects basing on the grasp type, both the geometry and the volume are considered [58, 59]. These two features influence the level of accuracy in grasping the object (precision/intermediate/power), the number of fingers involved, and the positioning of the thumb (adducted/abducted). Therefore, the influence of both the geometry and volume of objects, summarized in the grasp type, have an impact on the subjects’ motor difficulty, and not only the object’s geometry.

In this section, it has been shown that the manipulation of different objects affects the stochastic Index of Difficulty, and in particular, agents experience a different motor difficulty when manipulating objects with different grasp types. In the next section, further analysis has been performed to investigate how objects characterized by a given grasp type, affect the speed of execution and the stochastic width.

### Velocity profiles and stochastic width

**Fig 6:**
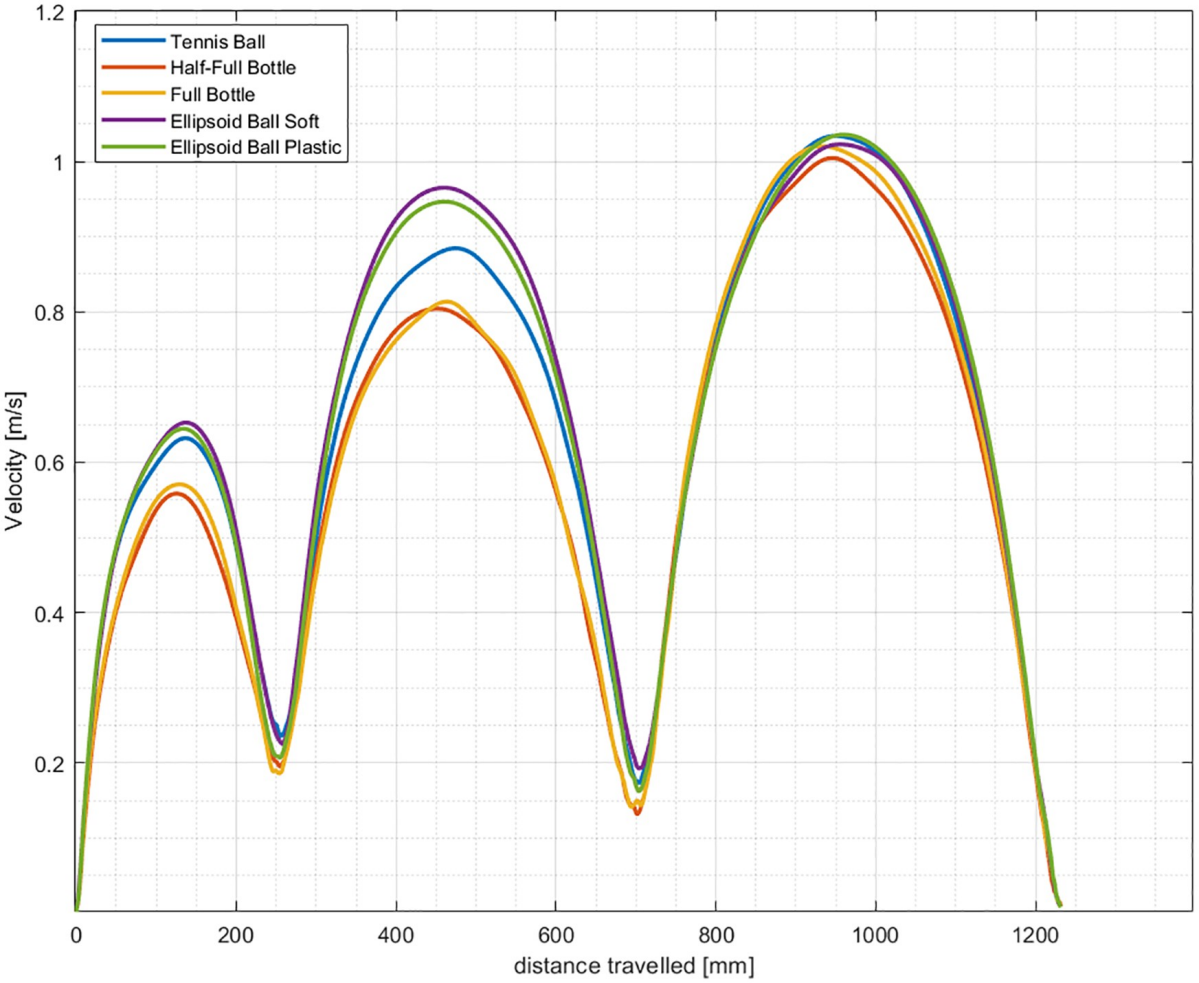
Velocity profiles values averaged over 31 subjects during the object manipulation task.

The velocity profiles related to the manipulation of the five objects are bell-shaped (Fig 6). Features of the velocity profiles are in line with the scientific literature since highest values are reached approximately halfway the path travelled [60, 61], with greater peaks for greater distances travelled [44, 62]. During the reaching and transport phases, the velocity peaks observed in the bell-shaped profiles are higher for the ellipsoid balls (soft and plastic) as compared to the tennis ball and bottles (full and half-full).

Differences in the velocity profiles are accentuated halfway the path of each phase, where the peak velocity is reached; these differences are not substantial during the return phase since subjects do not handle any object. Furthermore, in both the reaching and transport phases, it can be observed that the velocity profiles of the two bottles (half-full and full) and of the two ellipsoid balls (soft and plastic) are almost overlapping, confirming that neither the mass nor the stiffness influence the speed of execution (as well as difficulty, as observed before).

**Fig 7:**
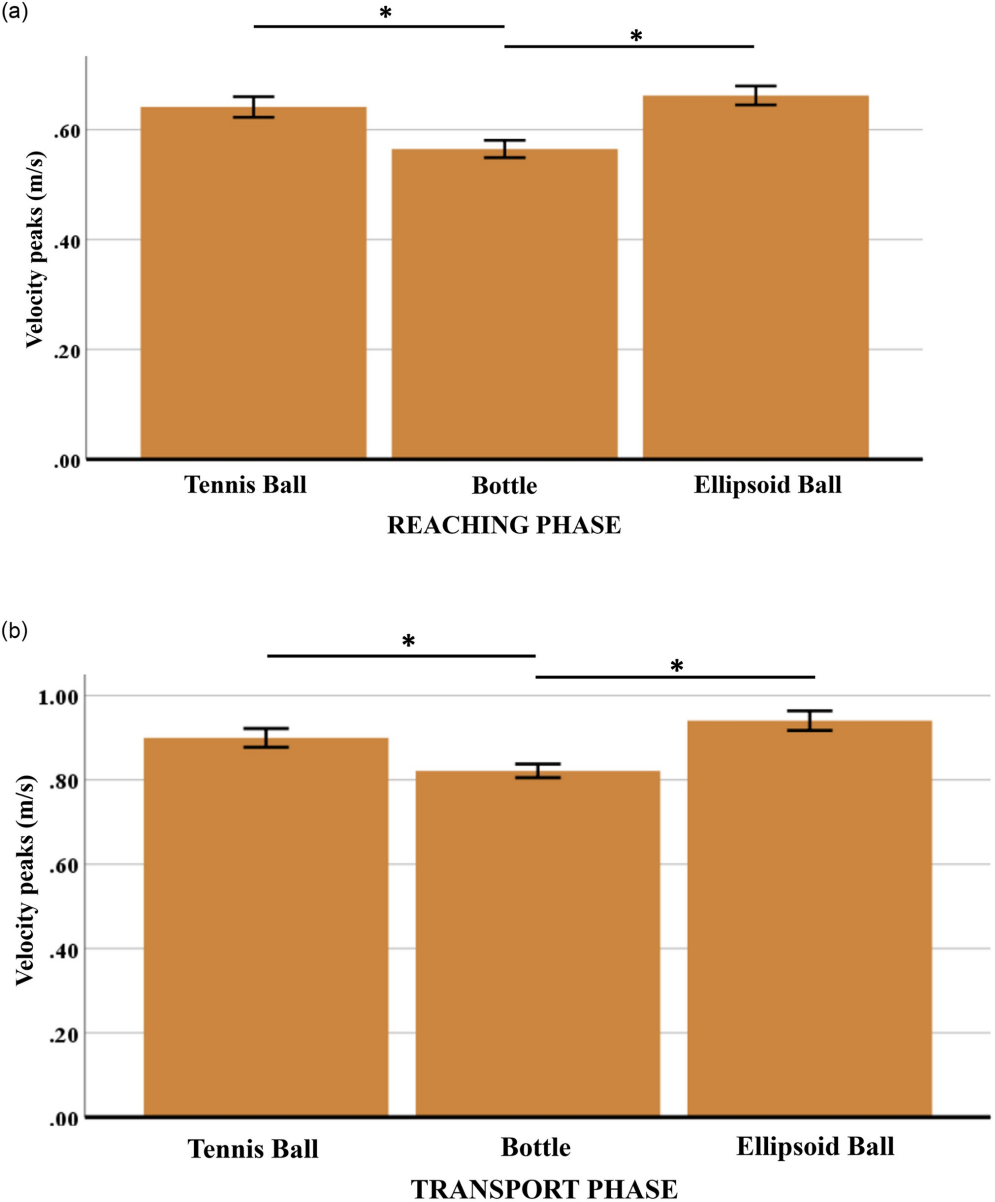
Mean values and SEM (Standard Error of the Mean) of velocity peaks at the reaching (7a) and transport (7b) phase for the three different geometrical objects. Asterisks indicate pairwise significant differences (p-value < 0.001); critical p-value = 0.05/3 = 0.016.

A statistical analysis of the velocity peaks for the three different geometrical objects has been carried out. The goal was to verify if the geometry alone or the grasp type have a significant effect on the velocity profiles. A one-way repeated-measures ANOVA has been conducted to evaluate if there is a statistically significant difference between the velocity peaks of the three different geometrical objects for the reaching and transport phases. After checking the assumptions prior to ANOVA (normality through Shapiro-Wilk test, sphericity through Mauchly’s test), results show statistically significant differences in the reaching (*F*(2,60) = 39.234, *p* < 0.0001) and transport phase (*F*(2,60) = 45.219, *p* < 0.0001). By applying the post hoc test with Bonferroni correction, the statistically significant difference of velocity peaks is evident in case of the ellipsoid/tennis ball vs. bottle (*p* < 0.001) in both the reaching and transport phases (Fig 7).

These results confirm that velocity peaks (and velocity profiles) of movements executed to reach, grasp, move and release an object are affected by the object-related grasp type. Again, the geometry of the object is not the only feature that influences movements performed, but also the volume. These two features together characterize the object’s grasp type [58, 59]. Moreover, by comparing Fig 6 with Fig 4, it can be observed that in presence of objects associated with a greater motor difficulty (e.g., bottles), velocity peaks and profiles are lower. This phenomenon is caused by the external constraints: higher motor difficulty implies that the agent’s motor system is more constrained (less flexible), resulting in reduced maximum speed and reachable configurations (exploitation of motor redundancy).

**Fig 8:**
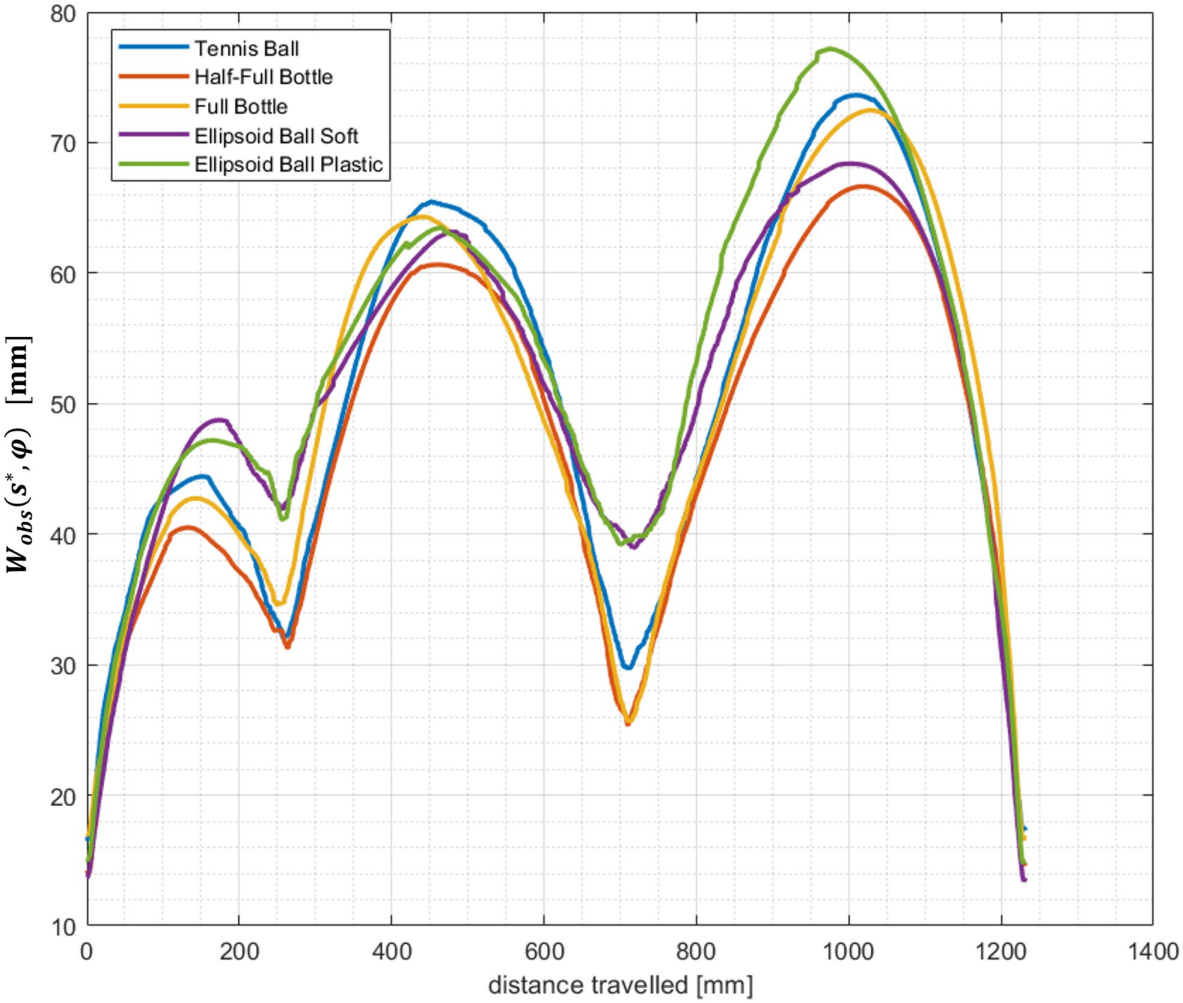
*W*_*obs*_(s*, *φ*) values averaged over 31 subjects during the object manipulation task.

The stochastic width profile *W*_*obs*_(s*, *φ*) for each given object (averaged over 31 subjects) is bell-shaped in each phase of the task, with smaller values where accuracy of the end-effector’s position is required (Fig 8).

The bell-shaped profiles of *W*_*obs*_(s*, *φ*) are consistent with the velocity ones: events that require greatest accuracy (i.e., smaller *W*_*obs*_(s*, *φ*) values) occur at the lowest speed values, and vice-versa. Furthermore, the maxima of both stochastic width and velocity profiles increase as the distance travelled within each phase increases (the distance travelled in the reaching phase is approximately half the one of the transport phase). A greater travelling distance allows the subjects to go faster (Fig 6) and attain a greater number of end-effector configurations (Fig 8).

## Conclusions

Starting from the original definition of the Index of Difficulty given by Fitts [5], the paper discussed a novel concept and proposed an analytical-stochastic model of experienced motor difficulty (*ID*_*obs*_) by considering the stochastic behaviour of agents performing a given repetitive motor task. The agent’s motor difficulty is no longer a deterministic function of task geometry, but it is indicative of the stochastic behaviour of an agent as it performs a motor task while interacting with the environment: the motor difficulty is not a unique characteristic of an agent, but it is the unique characteristic of the combined agent-environment system.

By applying the stochastic Index of Difficulty to the three-dimensional manipulation of different objects, a higher motor difficulty has been experienced by subjects when handling objects characterized by a “large diameter” grip (bottle), compared to objects characterized by a “power sphere” grip (ellipsoid/tennis ball); the different mass and stiffness of the considered objects, instead, did not influence the subject’s motor difficulty. Results obtained can help future work in which a complete factorial experimental analysis will be designed to investigate the effects of multiple objects’ features (mass, volume, stiffness, geometry, main axis of symmetry), and their interaction; the collection of a large dataset will be necessary, where the objects manipulated can be compared within and across multiple features, and agents with different motor abilities (e.g., age, experience) will be recruited. Nonetheless, the current dataset and analysis has provided useful information on what could be, among many, the main relevant factors in object manipulation tasks, hence the opportunity to formulate more refined hypotheses in the future about the effect of specific objects’ features on the motor difficulty.

The use of the stochastic Index of Difficulty, and the evaluation of the agent’s motor difficulty, is not limited to the specific application of manipulation tasks but can be employed in multiple fields of investigation. As an example, *ID*_*obs*_ can be used to design the layout of the workplace, such as in assembly workstations, since the kinematics of operators and motor strategies to be adopted to reach points are affected by the presence of obstacles, positions of targets, positions of components/parts to handle etc… [63, 64]. Furthermore, *ID*_*obs*_ can be a valuable tool to be employed in job rotation/resource allocation problems. As an example, by observing a reference operator that executes optimally a given motor task, its motor difficulty can be quantified; this information can be employed to compare the *ID*_*obs*_ of other operators and choose the operators whose motor behaviour it the closest to the ‘optimal’ one.

Further research will be focused in applying in industrial contexts the analytical-stochastic model proposed to analyse the motor difficulty of differently skilled operators in executing manual operations characterized by different motor complexity. Analogous research approaches can be translated to the context of motor rehabilitation.

## Abbreviations

$\bar{t}$: Average trajectory observed for each subject in each condition
$\chi_{\nu_{1},\varphi }^{2}$: Chi-squared distribution with *ν*_1_ degrees of freedom, at a given probability level
$F_{\nu_{1},\nu_{2},\varphi }$: F-distribution with *ν*_1_ degrees of freedom of the numerator and *ν*_2_ degrees of freedom of the denominator
{*x*, *y*, *z*}: Global reference system
{*ϵ*(*s*), *η*(*s*), *ψ*(*s*)}: Local reference system laying on *P*(*s*) with origin at *p*(*s*)
*c* ^2^: Constant statistical squared distance for the standard deviation ellipse
*ds*: Infinitesimal length of $\bar{t}$
*f*: Number of coordinates of the reference systems (*f* = 3)
*ID*: Index of Difficulty
*ID*_*obs*_(*s*, *φ*): Stochastic Index of Difficulty (observation-based) at the curvilinear coordinate *s* and probability level *φ*
*ID*_*t*_: Index of Difficulty for a pre-defined general planar trajectory *t*
*k*: Index for the *k*^*th*^ trajectory; 1 < *k* < *n*
*MD* ^2^: Squared Mahalanobis distance
*n*: Number of trajectories (i.e., trials) executed by each subject in each condition
*P*(*s*): Plane orthogonal to $\bar{t}$, at coordinate *s*
*P*(*s*): Point along $\bar{t}$, at coordinate *s*
*PCA*: Principal Component Analysis
*q*_*k*_(*s*): Intersection point between *t*_*k*_ and *P*(*s*)
*s*: Curvilinear coordinate of $\bar{t}$
*T* ^2^: Hotelling’s T-squared distance
*t*_*k*_: *k*^*th*^ spatial trajectory
*W*: Constant target width
*W*_*obs*_(*s*, *φ*): Stochastic width, representative of the variability of observed trajectories at the curvilinear coordinate *s* and probability level *φ*
*W*_*t*_(*s*): Target width at the curvilinear coordinate *s* of a general planar trajectory *t*
*σ*_*ϵ*_(*s*): Standard deviation of all *q*_*k*_(*s*) points, 1 < *k* < *n*, along the local *ϵ*-coordinate
*σ*_*η*_(*s*): Standard deviation of all *q*_*k*_(*s*) points, 1 < *k* < *n*, along the local *η*-coordinate
*φ*: Probability level
